# In Vitro and In Vivo Anti-Cancer Activity of Lasiokaurin in a Triple-Negative Breast Cancer Model

**DOI:** 10.3390/molecules28237701

**Published:** 2023-11-22

**Authors:** Jinrong Lin, Zhao Qu, Huanhuan Pu, Li-Sha Shen, Xianguo Yi, Yu-Shan Lin, Rui-Hong Gong, Guo-Qing Chen, Sibao Chen

**Affiliations:** 1 Institute of Medicinal Plant Development, Chinese Academy of Medical Sciences and Peking Union Medical College, Beijing 100193, China; 2 Third-Grade Pharmacological Laboratory on Chinese Medicine Approved by State Administration of Traditional Chinese Medicine, Medical College, China Three Gorges University, Yichang 443002, China; 3 Chongqing Academy of Chinese Materia Medica, Chongqing 400065, China; 4 College of Animal Science and Technology, Xinyang Agricultural and Forestry University, Xinyang 464000, China; yixianguo@126.com; 5 State Key Laboratory of Chinese Medicine and Molecular Pharmacology (Incubation), The Hong Kong Polytechnic University Shenzhen Research Institute, Shenzhen 518057, China; 6 Department of Food Science and Nutrition, The Hong Kong Polytechnic University, Hung Hom, Hong Kong 999077, China; 7 Research Centre for Chinese Medicine Innovation, The Hong Kong Polytechnic University, Hung Hom, Hong Kong 999077, China

**Keywords:** lasiokaurin, isodon, triple-negative breast cancer, PI3K/Akt/mTOR, STAT3

## Abstract

Due to its intricate heterogeneity, high invasiveness, and poor prognosis, triple-negative breast cancer (TNBC) stands out as the most formidable subtype of breast cancer. At present, chemotherapy remains the prevailing treatment modality for TNBC, primarily due to its lack of estrogen receptors (ERs), progesterone receptors (PRs), and human epidermal growth receptor 2 (HER2). However, clinical chemotherapy for TNBC is marked by its limited efficacy and a pronounced incidence of adverse effects. Consequently, there is a pressing need for novel drugs to treat TNBC. Given the rich repository of diverse natural compounds in traditional Chinese medicine, identifying potential anti-TNBC agents is a viable strategy. This study investigated lasiokaurin (LAS), a natural diterpenoid abundantly present in Isodon plants, revealing its significant anti-TNBC activity both in vitro and in vivo. Notably, LAS treatment induced cell cycle arrest, apoptosis, and DNA damage in TNBC cells, while concurrently inhibiting cell metastasis. In addition, LAS effectively inhibited the activation of the phosphatidylinositol-3-kinase/protein kinase B/mammalian target of rapamycin (PI3K/Akt/mTOR) pathway and signal transducer and activator of transcription 3 (STAT3), thus establishing its potential for multitarget therapy against TNBC. Furthermore, LAS demonstrated its ability to reduce tumor growth in a xenograft mouse model without exerting detrimental effects on the body weight or vital organs, confirming its safe applicability for TNBC treatment. Overall, this study shows that LAS is a potent candidate for treating TNBC.

## 1. Introduction

Breast cancer stands as the predominant form of solid malignant tumors and is the leading contributor of cancer-related mortality among women worldwide [1]. Triple-negative breast cancer (TNBC), classified as a subtype within the realm of breast cancer, is identified via immunohistochemistry by the lack of estrogen receptors (ERs), progesterone receptors (PRs), and human epidermal growth receptor 2 (HER2). This subtype constitutes approximately 15–20% of the total breast cancer cases [2]. TNBC displays a greater prevalence among young and pre-menopausal women [3,4]. Alongside its elevated risk of relapse and metastasis, TNBC is characterized by a notably brief progression-free survival period and a low overall survival rate. Because of the absence of ERs, PRs, and HER2, TNBC does not respond to endocrine and anti-HER2 therapies. Currently, unresectable TNBC is treated with non-targeted chemotherapeutic agents, such as paclitaxel and anthracyclines. Regrettably, cytotoxic chemotherapy is associated with the emergence of various undesirable side effects [5,6]. Thus, an urgent imperative exists to develop novel drugs with heightened selectivity and reduced side effects for TNBC treatment.

**Figure 1:**
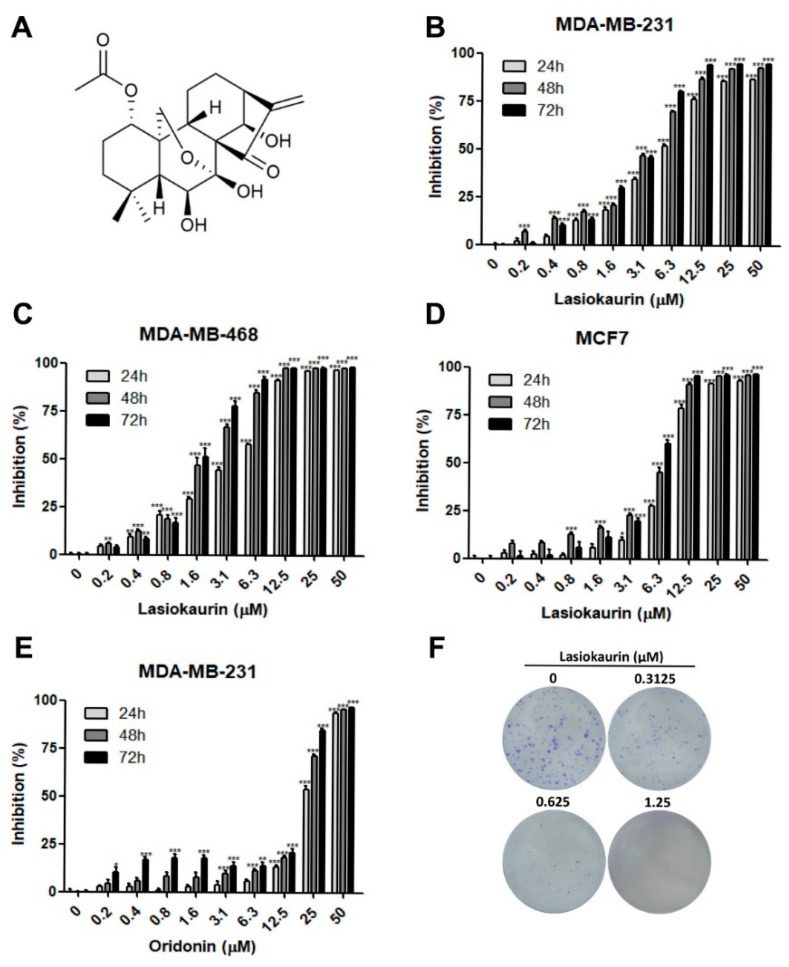
LAS inhibited breast cancer cell proliferation. (**A**) Chemical structure of LAS. (**B**–**D**) Cell viability of MDA-MB-231, MDA-MB-468, and MCF7 was separately measured by MTT assay after LAS treatment. (**E**) Cell viability of MDA-MB-231 was measured by MTT assay after oridonin treatment. (**F**) Colony formation ability of MDA-MB-231 cells treated with LAS for 13 days. Data are presented as means ± SEM from three independent experiments. * *p* < 0.05, ** *p* < 0.01, *** *p* < 0.001, compared to control.

Owing to their substantial abundance and wide-ranging chemical structural diversity, natural products derived from certain traditional medicines have gained widespread recognition for their application in addressing a variety of diseases, including TNBC [7,8,9,10,11]. Belonging to the Lamiaceae family, the genus *Isodon* encompasses a cluster of flowering plants and comprises approximately 100 species, predominantly found across tropical and subtropical regions of Asia [12]. Within the *Isodon* genus, plants harbor an extensive array of diterpenoids with multifaceted biological functions, including anti-cancer, anti-inflammatory, and anti-viral properties [13,14]. Among the diterpenoids in *Isodon* plants, oridonin stands out as a notable example, drawing growing interest for its extensive range of anti-cancer activities [15]. Previous studies demonstrated that oridonin exhibited anti-cancer effects against breast cancer [16,17,18], colon cancer [19,20], lung cancer [21], nasopharyngeal carcinoma [22], oral cancer [23], pancreatic cancer [24], bladder cancer [25], and neuroblastoma [26]. However, lasiokaurin (LAS; Figure 1A), a significant analogue of oridonin in *Isodon* plants, has not yet attracted much attention from the research community. Only a limited number of earlier studies have noted LAS’s anti-microbial and anti-tumor activities [27,28]. Nevertheless, the exploration of LAS’s potential as an anti-TNBC agent remains largely uncharted territory. In light of this gap, the present study is dedicated to investigating LAS’s anti-cancer effects on TNBC, along with the underlying mechanisms.

## 2. Results

### 2.1. LAS Inhibits the Proliferation of TNBC Cells

To evaluate the in vitro anti-cancer activity of LAS in breast cancer, two TNBC cell lines, namely MDA-MB-231 and MDA-MB-468, along with the ER and PR positive cell line MCF7, were employed. Additionally, the MCF-10A cell line, serving as a model for normal human breast cells, was utilized to analyze the cytotoxicity of LAS. The MTT assay results demonstrated a notable dose- and time-dependent reduction in cell viability with increasing concentrations of LAS (0.2–50 μM), as depicted in Figure 1B–D. Notably, LAS exhibited an inhibitory effect on both breast cancer cells and normal breast cells, although the impact on normal cells was comparatively weaker (Figure S1). Furthermore, we explored the impact of oridonin on MDA-MB-231 cell viability (Figure 1E), and the IC_50_ values for LAS and oridonin were separately presented in Table 1. These findings collectively highlight that LAS was more potent in diminishing TNBC cell viability compared to oridonin. Additionally, LAS demonstrated a relatively low level of toxicity to normal cells. To assess the extended inhibitory impact of LAS on TNBC cells, colony formation assays were conducted. As expected, even at a low and non-toxic concentration of LAS (0.3125 μM), the colony formation ability of MDA-MB-231 cells was notably suppressed (Figure 1F). These findings collectively indicated that LAS exhibited the potential to inhibit TNBC cell proliferation and survival in vitro.

### 2.2. LAS Modulates Cell Cycle Progression in TNBC Cells

**Figure 2:**
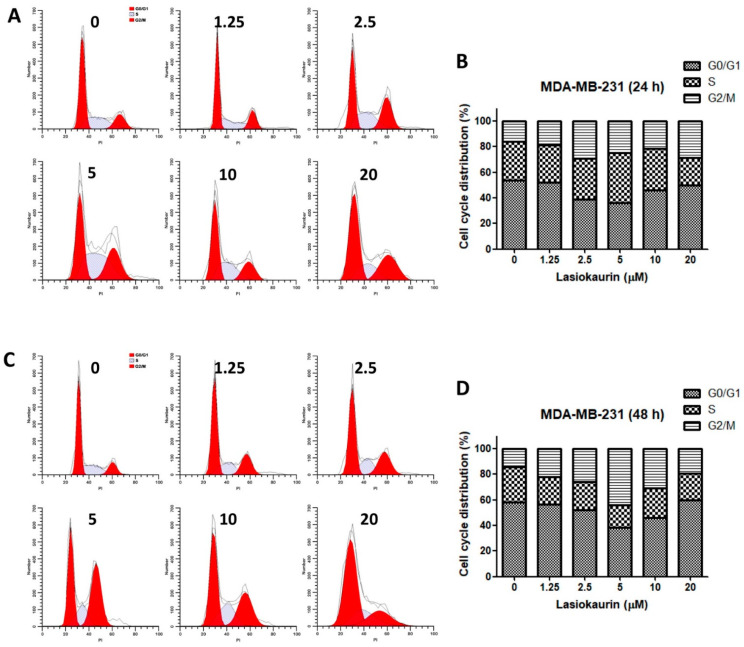
LAS induced cell cycle arrest in MDA-MB-231 cells. MDA-MB-231 cells were stained with PI after LAS treatment and the cell cycle analyzed by flow cytometry. Representative DNA fluorescence histograms of cell cycle distribution after 24 h (**A**) and 48 h (**C**) treatment were presented. Bar charts showed the percentage of different phases after 24 h (**B**) and 48 h (**D**) treatment.

The progression of the cell cycle plays a crucial role in determining cell proliferation outcomes [29]. Therefore, the current study focused on the regulation of cell cycle progression to uncover the potential underlying mechanisms of LAS. Cell cycle distribution in the LAS-treated MDA-MB-231 and MDA-MB-468 cells was evaluated by PI staining using flow cytometric analysis. Figure 2A–D shows the effect of the rising LAS concentration (1.25–20 μM) on the MDA-MB-231 cell cycle. Remarkably, LAS led to a dose- and time-dependent induction of cell cycle arrest specifically at the G2/M phase. Interestingly, this arrest effect saw a decline in cells treated with LAS concentrations exceeding 5 μM. Parallel observations were made in the LAS-treated MDA-MB-468 cells (Figure S2A–D). The outcomes of the aforementioned experiments collectively indicated that G2/M phase cell cycle arrest in TNBC cells was notably induced only with low LAS concentrations, and other mechanisms could be involved in LAS concentrations exceeding 5 μM.

### 2.3. LAS Induces Apoptosis and DNA Damage in TNBC Cells

**Figure 3:**
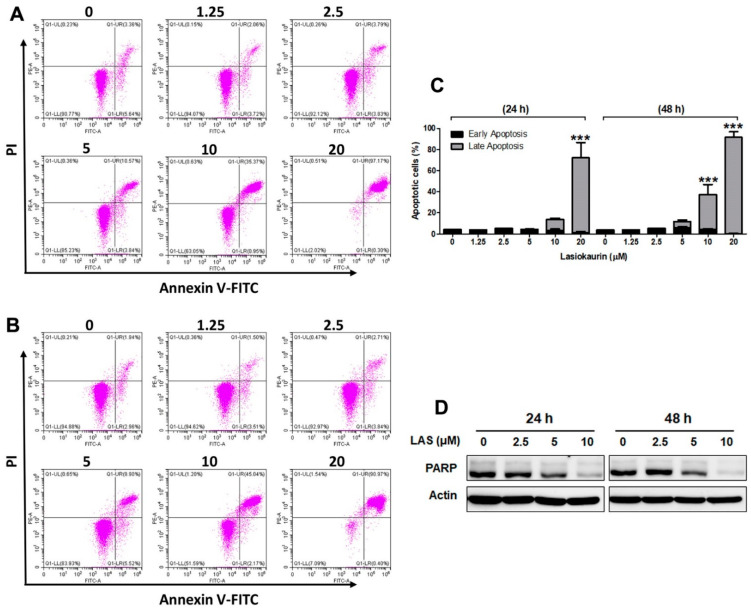
LAS induced apoptosis and DNA damage in MDA-MB-231 cells. MDA-MB-231 cells were treated with LAS for 24 h (**A**) and 48 h (**B**), stained with Annexin V-FITC/PI, and cell apoptosis was analyzed by flow cytometry. (**C**) Representative flow cytometry Annexin V/PI data. *** *p* < 0.001, compared to control. (**D**) Cell extracts were prepared from MDA-MB-231 cells and immunoblotted with the indicated antibodies. β-Actin was used as an internal control.

In addition to cell cycle regulation, cell death activation is also considered as an alternative strategy for cancer therapy [30]. Apoptosis is a major form of programmed cell death, while several natural compounds have been identified to promote apoptosis in cancer cells [31]. Herein, to explore the effect of LAS on inducing cell apoptosis, Annexin V-FITC/PI staining was performed via flow cytometry. Figure 3A–C reveals that lower LAS concentrations (1.25 μM and 2.5 μM) did not induce cell apoptosis in MDA-MB-231 cells. Interestingly, the landscape shifted when TNBC cells were exposed to 5–20 μM LAS for 24 and 48 h, resulting in a substantial rise in the proportion of apoptotic cells. Remarkably, the consistent results were mirrored in the LAS-treated MDA-MB-468 cells (Figure S3A–C). The collective evidence underscored that LAS concentrations exceeding 5 μM exerted a marked induction of cell apoptosis.

DNA damage is considered as a significant strategy for killing cancer cells. Therefore, the expression levels of PARP, which is involved in repairing DNA damage [32], were detected. Figure 3D reveals that LAS significantly suppressed PARP expression in MDA-MB-231 cells, suggesting LAS’s potential to induce DNA damage in TNBC cells. Correspondingly, consistent outcomes emerged in the LAS-treated MDA-MB-468 cells (Figure S3D). The above results demonstrated that treating TNBC cells with lower LAS concentrations predominantly triggered cell cycle arrest, whereas higher LAS concentrations induced both cell apoptosis and DNA damage.

### 2.4. LAS Inhibits the Migration and Invasion of TNBC Cells

**Figure 4:**
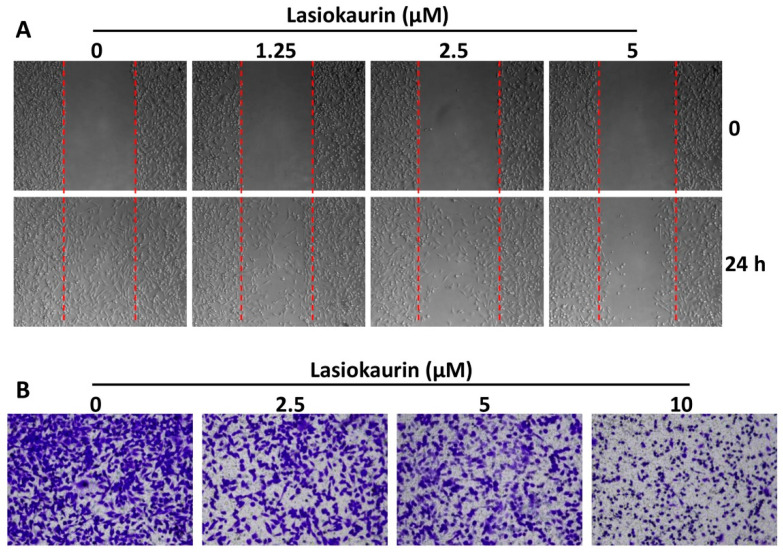
LAS inhibited the migration and invasion of MDA-MB-231 cells. (**A**) Cell migration was measured by wound-healing assay. (**B**) Cell invasion ability was assessed by transwell invasion assay.

As the most aggressive form of breast cancer, TNBC exhibits heightened metastasis rates, inevitably leading to elevated mortality rates [33]. Therefore, an anti-TNBC compound with good potential should have anti-metastatic properties [34]. To determine whether LAS could affect the migration and invasion abilities of TNBC cells, wound-healing and transwell invasion assays were performed. As shown in Figure 4A, cell treatment with LAS for 24 h post-wounding remarkably dampened the migratory capacity of MDA-MB-231 cells in a dose-dependent manner. Correspondingly, LAS exerted a dose-dependent inhibition on the invasive potential of MDA-MB-231 cells (Figure 4B). Similarly, LAS’s impact on restraining migration and invasion was echoed in MDA-MB-468 cells (Figure S4A,B). Collectively, these findings suggested that LAS could inhibit TNBC cell metastasis in vitro.

### 2.5. LAS Inhibits PI3K/Akt/mTOR and STAT3 Signalling in TNBC Cells

**Figure 5:**
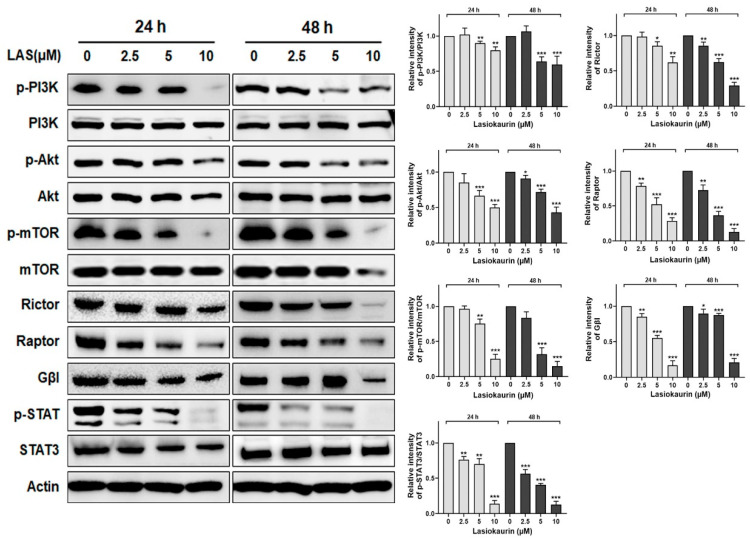
LAS inhibited PI3K/Akt/mTOR pathway and STAT3 in MDA-MB-231 cells. MDA-MB-231 cells were treated with LAS at concentrations of 2.5, 5, 10 μM for 24 or 48 h. Cell pellets collected and immunoblotted with the indicated antibodies. β-Actin was used as an internal control. Quantitative analysis of protein expression was in the right panel. * *p* < 0.05, ** *p* < 0.01, *** *p* < 0.001, compared to control.

The PI3K/Akt/mTOR pathway is one of the most significant and active pathways which are involved in TNBC development [35]. This pathway is known to play a pivotal role in regulating various cellular processes, including cell growth, proliferation, and metastasis [36]. Given its significance, inhibiting the PI3K/Akt/mTOR pathway holds promise as a therapeutic strategy for TNBC [37]. In this context, we investigated whether LAS could inhibit this pathway. Figure 5 shows a significant reduction in the phosphorylation levels of PI3K, Akt, and mTOR upon LAS treatment in MDA-MB-231 cells. Additionally, it is widely known that mTOR acts via multiprotein complexes, such as mTORC1 and mTORC2 [38], which interplay with the PI3K/Akt pathway. To discern the complex responsible for LAS’s inhibitory effect on TNBC, we evaluated the expression of Rictor, Raptor, and Gβl, three partners that can bind to mTOR [39]. The results demonstrated decreased levels of Rictor, Raptor, and Gβl in the LAS-treated MDA-MB-231 and MDA-MB-468 cells, thus affirming the inhibition of both mTORC1 and mTORC2 activities (Figure 5 and Figure S5).

Additionally, it has been reported that the transcription factor STAT3 is upregulated and constitutively activated in TNBC [40]. Prior research further demonstrated that STAT3 activation plays a crucial role in cancer cell proliferation, invasion, and migration [41], suggesting its potential as a therapeutic target for TNBC treatment. Consequently, we investigated the impact of LAS on STAT3 within TNBC cells. Our findings revealed that the expression levels of both STAT3 and p-STAT3 significantly decreased in a dose-dependent manner following LAS treatments at 24 h and 48 h (Figure 5 and Figure S5).

### 2.6. LAS Inhibits Tumor Growth in a Xenograft Nude Mouse Model

**Figure 6:**
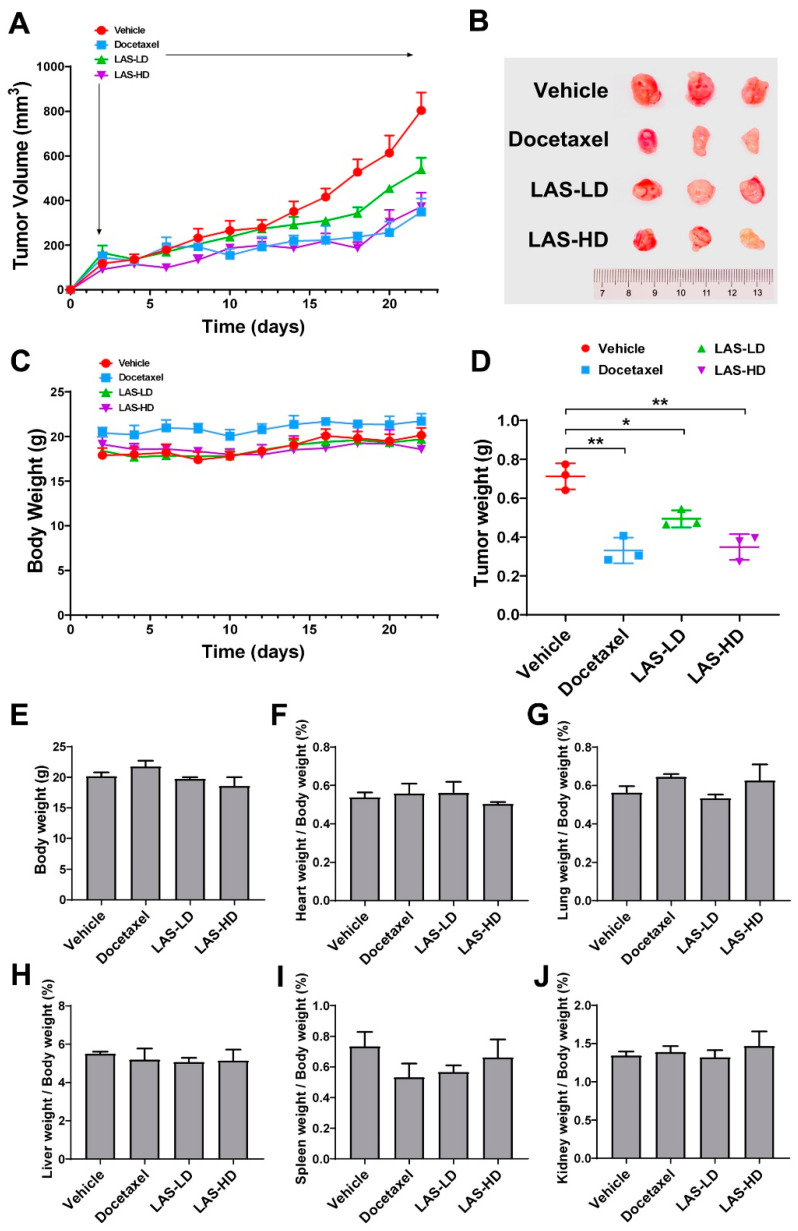
LAS inhibited in vivo MDA-MB-231 xenograft tumor growth. A xenograft model was established by subcutaneous inoculation of MDA-MB-231 cells into BALB/c nude mice mammary fat pads. When the average tumor volumes reached 120 mm3, mice were randomly divided into four groups and administrated with vehicle (5% of Cremophor EL, 5% of ethanol in saline), LAS-LD (5 mg/kg), or LAS-HD (10 mg/kg) daily, or docetaxel (10 mg/kg) via i.p. injection. The treatment period lasted for 20 days and all mice were sacrificed. (**A**) Tumor volumes were measured throughout the experimental period. (**B**) Images of tumors at the end of experiment. (**C**) Mouse body weights throughout the experimental period. (**D**) Tumor weights at experimental endpoint. (**E**–**J**) Organ weights normalized to body weights and expressed at the percentage of body weight. Data are expressed as means ± SEM.* *p* < 0.05, ** *p* < 0.01, compared to the vehicle group.

**Figure 7:**
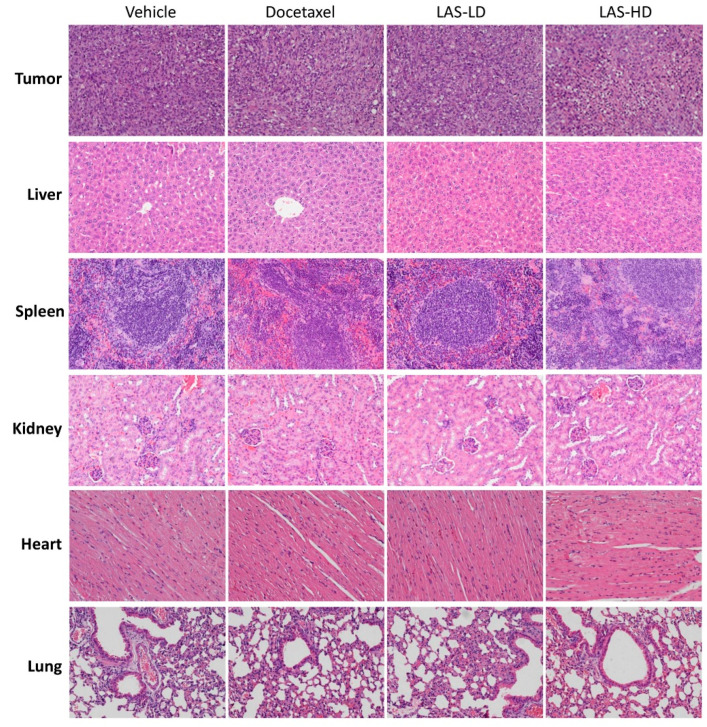
Photomicrograph of H&E histology of tissues (tumor, liver, spleen, kidney, heart, lung) after LAS treatment. Representative images are shown (20×).

To investigate the in vivo anti-cancer effect of LAS, a mouse xenograft model was established via inoculating MDA-MB-231 cells into mammary fat pads. Following tumor inoculation, the mice received 5 mg/kg (LD group) or 10 mg/kg (HD group) LAS intraperitoneally daily for 20 consecutive days. Mice in the vehicle and positive control groups were also intraperitoneally injected with saline and docetaxel (10 mg/kg), respectively. After 20 days, the mice were sacrificed and the xenograft tumors and body organs were resected for evaluation. As shown in Figure 6A–D, the tumor volume and weight were significantly reduced in the LAS-LD and LAS-HD groups, thus indicating that LAS retarded tumor growth. While LAS-LD exhibited slightly lower efficacy than docetaxel, LAS-HD at the same dose showed comparable efficacy to docetaxel. Moreover, LAS had no discernible impact on the body weight, signifying its safety for application. In order to validate the aforementioned findings, we assessed the weight and histopathological attributes of the hearts, lungs, livers, spleens, and kidneys in mice from each group. As shown in Figure 6E–J and Figure 7, in comparison to the vehicle group, there were no noteworthy alterations in the organ weight or histopathological characteristics observed in either the LAS-treated groups or the vehicle group. Collectively, these findings indicated that LAS could suppress tumor growth in vivo without inducing discernible toxicity.

## 3. Discussion

Clinically, TNBC stands as the most aggressive subtype of breast cancer, characterized by high recurrence and metastasis rates. Owing to the absence of relevant receptor markers, patients with TNBC derive limited benefits from conventional targeted chemotherapies. Consequently, despite the adversities of its side effects, non-specific chemotherapy remains the established treatment approach for TNBC patients. Therefore, the imperative pursuit of novel, effective TNBC treatments devoid of side effects assumes paramount significance.

In recent years, a range of innovative treatment strategies has emerged, encompassing groundbreaking concepts like artificial intelligence (AI). Despite these advancements, natural compounds are still the most significant sources of novel drugs. Several anti-cancer drugs, such as paclitaxel, vinblastine, and camptothecin, were born out of the screening of natural compounds and have been widely used in clinical practice.

In our present study, we investigated the anti-TNBC activity of LAS, a natural diterpenoid discovered within the *Isodon* genus, serving as an analogue to oridonin. We initiated our exploration with MTT and colony formation assays, revealing the pronounced inhibitory effects of LAS on the proliferation of TNBC cells. To elucidate the potential anti-cancer mechanism of LAS in TNBC, we investigated the distribution of the cell cycle. The results showed that LAS, at lower doses, significantly induced cell cycle arrest in the G2/M phase. Interestingly, as the LAS concentrations increased, its efficacy in inducing cell cycle arrest diminished. This observation suggests that the primary mechanism driving the inhibitory impact of low LAS concentrations on TNBC is cell cycle arrest. Furthermore, additional mechanisms might come into play as LAS concentrations rise. Subsequently, we assessed LAS-induced apoptosis in TNBC cells using Annexin V-FITC/PI staining with flow cytometry. The findings demonstrated a dose-dependent increase in the proportion of apoptotic cells with rising LAS concentrations. Given the effectiveness of DNA damage response in treating TNBC, we further investigated whether LAS could induce DNA damage in TNBC cells [42]. The results indicated that LAS could trigger DNA damage in TNBC cells by inhibiting PARP expression, an enzyme crucial for DNA damage repair [43]. Collectively, these outcomes confirm the hypothesis: low LAS concentrations facilitate TNBC cell cycle arrest in the G2/M phase, while higher concentrations induce TNBC cell apoptosis and DNA damage.

In clinical practice, a potential therapeutic strategy for TNBC involves inhibiting the migration and invasion abilities of TNBC cells [44]. Consequently, an effective anti-TNBC compound should possess anti-metastatic properties as well. With this context in mind, we proceeded to investigate the impact of LAS on inhibiting the migration and invasion capabilities of TNBC cells. In addition to its observed effect in suppressing cell migration during the wound-healing assay, LAS also demonstrated the ability to curtail the invasive potential of TNBC cells in transwell invasion assays. This combined evidence suggests that LAS holds promise in restraining TNBC metastasis.

It has been reported that the PI3K/Akt/mTOR pathway is overactivated in over 60% of patients with TNBC, eventually contributing to cancer cell proliferation, metastasis, and survival [45]. Previous research demonstrated a correlation between the expression of p-mTOR and unfavorable prognosis in early-stage TNBC patients [46]. In recent years, various inhibitors targeting the PI3K/Akt/mTOR pathway have undergone clinical trials. Examples include PQR309, a dual PI3K/mTOR inhibitor, as well as Ipatasertib and AZD5363, both Akt inhibitors, and temsirolimus, an mTOR inhibitor [37]. However, these endeavors have yielded less-than-optimal outcomes, underscoring the complexity of treating multifaceted conditions like TNBC with single-target chemotherapy. This scenario has prompted a shift toward multitarget therapeutics for TNBC treatment, a strategy that has exhibited enhanced effectiveness [47]. For example, promising results have emerged from studies targeting both mTOR and STAT3 in TNBC treatment [48]. Furthermore, it is worth noting that the transcription factor STAT3 is frequently overexpressed and constitutively active in TNBC, thus holding a crucial role in anti-TNBC strategies [40]. The present study showed that LAS not only inhibited the activation of the PI3K/Akt/mTOR pathway but also dampened that of STAT3. This observation indicates LAS can be a potential multitarget therapeutic candidate for TNBC.

The inhibitory effects of LAS on tumor growth were evaluated in a subcutaneous TNBC xenograft mouse model. Remarkably, even at low doses, LAS significantly curtailed tumor growth in vivo. Furthermore, the escalating doses of LAS exhibited a similar inhibitory effect to that of docetaxel, one of the most efficacious chemotherapy drugs for TNBC. Importantly, LAS administration did not result in weight loss, abnormal behavior, or histopathological changes in critical organs. These observations underscore the safety of applying LAS in TNBC treatment.

## 4. Materials and Methods

### 4.1. Compounds

LAS (C_22_H_30_O_7_; cat. no. CAS28957-08-6) was purchased from Jiangsu Yongjian Pharmaceutical Technology Co., Ltd. (Yangzhou, China), while oridonin (C_20_H_28_O_6_; cat. no. CAS28957-04-2) was purchased from Shanghai Aladdin Biochemical Technology Co., Ltd. (Shanghai, China) The purity of both compounds was >98% as analyzed by high-performance liquid chromatography.

### 4.2. Cell Culture

The human TNBC cell lines, MDA-MB-231 and MDA-MB-468, and the non-TNBC cell line, MCF7, were purchased from the American Type Culture Collection (ATCC, Manassas, VA, USA). All cell lines were maintained in DMEM supplemented with 10% heat-inactivated FBS and 1% penicillin/streptomycin solution at 37 °C in a humidified incubator with 5% CO_2_. The cell lines were used within two months after resuscitation and mycoplasma contamination was assessed utilizing the PCR Mycoplasma Detection Kit (Beijing Transgen Biotech Co., Ltd., Beijing, China).

### 4.3. Cell Viability Assay

A 3-(4, 5-dimethylthiazol-2-yl)-2,5-diphenyltetrazolium bromide (MTT) assay was used to investigate the effects of LAS and oridonin on breast cancer cell viability according to the report [49]. Briefly, following incubation for 24 h, cells were seeded in 96-well plates at a density of 5 × 103 cells/well (MDA-MB-231 and MCF7) or 1 × 10^4^ cells/well (MDA-MB-468) and were then treated with different concentrations of LAS or oridonin for an additional 24, 48, or 72 h. Untreated cells served as the control group. Subsequently, cells were treated with 0.5 mg/mL MTT (MilliporeSigma, Burlington, MA, USA) and incubated for 4 h at 37 °C. After discarding culture media, the wells were supplemented with DMSO to dissolve the formed formazan. The optical density (OD) at a wavelength of 570 nm was measured using a Biotek Synergy H1 microplate reader (BioTek Instruments, Inc., Winooski, VT, USA). The experiments were performed in six parallel wells and repeated for three times. The half-maximal inhibitory concentration (IC_50_) values were calculated using Graphpad Prism 5 software (GraphPad Software Inc., La Jolla, CA, USA).

### 4.4. Colony Formation Assay

According to a method in the publication [50], MDA-MB-231 cells were seeded in triplicate in 6-well plates at a density of 600 cells/well and were then treated with different concentrations (0, 0.3125, 0.625, and 1.25 µM) of LAS. Following culturing for 13 days, when the colonies were visible, the cell culture was terminated. Subsequently, cells were fixed with anhydrous methanol for 5 min, dried, and stained with 0.1% crystal violet solution for 10 min at room temperature. The excess dyes were washed away with MilliQ water, the formed colonies were dried, and images were then captured.

### 4.5. Cell Cycle Assay

Cell cycle distribution was assessed using the Cell Cycle and Apoptosis Analysis kit (Beyotime Biotechnology, Pudong, China). In accordance with the report [51], MDA-MB-231 and MDA-MB-468 cells were seeded into 6-well plates and treated with LAS for 24 or 48 h. Subsequently, cells were harvested, fixed with ice-cold 70% ethanol at 4 °C overnight, rinsed in PBS, and were then incubated with propidium iodide (PI) and RNase for 30 min at 37 °C in the dark. Flow cytometric analysis was performed using the BD AccuriC6 flow cytometry system (Becton Dickson Immunocytometry-Systems, San Diego, CA, USA) and the cell cycle distribution was analyzed using ModFit LT 5.0 (Verity Software House, Topsham, ME, USA).

### 4.6. Annexin V-FITC Apoptosis Assay

Cell apoptosis assay was carried out using the Annexin V-FITC Apoptosis Detection kit (Beyotime Biotechnology). Using a method in the report [52], MDA-MB-231 and MDA-MB-468 cells were seeded into 6-well plates and were then treated with LAS for 24 or 48 h. Following digestion with 0.25% trypsin (without EDTA), cells were harvested and incubated with 195 μL binding buffer supplemented with 5 µL FITC-labelled Annexin V and 10 µL PI for 20 min in the dark at room temperature. The fluorescence of cells was immediately quantified on the CytoFLEX flow cytometer (Beckman Coulter, Inc., Brea, CA, USA).

### 4.7. Wound-Healing Assay

The migration ability of TNBC was evaluated by wound-healing assays, as previously described [53]. Briefly, MDA-MB-231 cells at a density of 4 × 10^4^ cells/well were seeded into both chambers of the culture insert (Ibidi GmbH, Gräfelfing, Germany). After allowing cells to attach overnight, the inserts were removed to create a wound. The cells were then washed with serum-free medium to remove non-adherent cells, followed by treatment with various concentrations of LAS in 2% FBS-containing medium. Images of the migrated cells were captured under a microscope at 0 and 24 h in the same three randomly selected fields.

### 4.8. Transwell Invasion Assay

Transwell invasion assay was performed as previously reported using Corning transwell insert chambers with a pore size of 8 μm [50]. MDA-MB-231 and MDA-MB-468 cells were seeded at a density of 5 × 10^4^ cells/chamber in 200 μL serum-free DMEM in the upper Matrigel-coated chamber of the transwell insert. The lower chamber was supplemented with 10% FBS medium (500 μL) as a chemo-attractant. Following incubation for 24 h at 37 °C, cells on the upper surface of the membrane were carefully removed with a cotton swab. Cells that had invaded to the lower surface of the membrane were fixed with 4% polyformaldehyde for 15 min and stained with crystal violet for 10 min. Following washing with MilliQ water, the membrane was air-dried and cells were counted under a light microscope (magnification, 10×).

### 4.9. Western Blot Analysis

Following cell treatment with different concentrations of LAS for 24 and 48 h, cells were harvested for western blot analysis. Cell pellets were firstly lysed in RIPA buffer and the protein concentration was measured using the Pierce™ BCA Protein Assay (Thermo Fisher Scientific, Inc., Waltham, MA, USA). Equal amounts of protein extracts were separated by SDS-PAGE and were then transferred onto PVDF membranes (Bio-Rad Laboratories, Inc., Hercules, CA, USA). The membranes were blocked with 5% non-fat milk followed by incubation with primary antibodies in 5% BSA at 4 °C overnight. Following washing with TBS-Tween-20 buffer, blots were incubated with the corresponding secondary antibodies. The primary antibodies used were the following: anti-poly (ADP ribose) polymerase (PARP; cat. no. ab191217), anti-phosphorylated (p)-phosphatidylinositol-3-kinase (PI3K; cat. no. ab182651), anti-p-mammalian target of rapamycin (mTOR; cat. no. ab137133), anti-protein kinase B (Akt; cat. no. ab179463), anti-Raptor (cat. no. ab26264), anti-Rictor (cat. no. ab70374), anti-G protein beta subunit like (Gbl; cat. no. ab228832), anti-STAT3 (cat. no. ab68153), anti-p-STAT3 (cat. no. ab76315; all from Abcam, 1:1000 dilution), PI3K (cat. no. 3011), mTOR (cat. no. 9964T; both from Cell Signaling Technology, Inc., 1:1000 dilution), p-Akt (cat. no. 66444-1; ProteinTech Group, Inc. (Rosemont, IL, USA), 1:1000 dilution), and β-actin (ZSGB-Bio, 1:5000 dilution).

### 4.10. Establishment of Tumor Xenograft Model

Female BALB/c nude mice were obtained from ZhuHai Bestest Biotechnology Co., Ltd. (Zhuhai, China). Mice were housed at room temperature (23 ± 2 °C) with a 12 h light/dark cycle and were given ad libitum access to food and water. All animal experiments were carried out at the Hong Kong Polytechnic University, according to the protocol approved by the Animal Subjects Ethics Sub-committee. Mice were inoculated with 5 × 10^6^ MDA-MB-231 cells suspended in 0.1 mL DMEM without FBS or penicillin/streptomycin, at the fourth mammary fat pad. When the average tumor volume reached 120 mm^3^, mice were randomly divided into four groups (*n* = 3 mice/group). Groups were balanced for mean tumor size. The treatment groups were as follows: Mice in the vehicle/negative group were injected intraperitoneally daily with an equivalent amount of solvent (5% Cremophor EL and 5% ethanol in saline; 10 mL/kg). Mice in the positive control group were treated with 10 mg/kg docetaxel, while those in the LAS group with 5 [LAS-Low Dose (LD)] or 10 mg/kg [LAS-High Dose (HD)] LAS. The body weight and tumor size of the mice were measured every other day. Tumor volume was calculated using the following formula: volume = (length × width^2^)/2. When the average tumor volume in the vehicle group reached ~800 mm^3^ (the maximum tumor volume reached 1094 mm^3^) on day 20 post-treatment, the experiment was terminated. The mice were euthanized by CO_2_ inhalation at a displacement rate of 50% cage volume per minute in a cage with 10 L for 2 min. After confirming the death of the mice, their tumors and vital organs, including heart, liver, spleen, lung, and kidney, were harvested and weighed.

### 4.11. Haematoxylin and Eosin (H&E) Staining

Tumors and vital organs were fixed in 10% neutral buffered formalin for three days and the tissues were then embedded in paraffin. Subsequently, the paraffin-embedded tissues were cut into 5 μm sections, followed by staining with H&E. Images of the H&E-stained sections were captured under a light microscope (magnification, 20×).

### 4.12. Statistical Analysis

All data were expressed as the mean ± SEM. The results were analyzed using SPSS 20.0 statistical software. The differences among multiple groups were compared by one-way ANOVA, followed by LSD post hoc or Dunnett’s tests. *p* < 0.05 was considered to indicate a statistically significant difference.

## 5. Conclusions

At present, there exists a scarcity of clinically efficacious pharmaceuticals employed in the treatment of TNBC. Despite the utilization of conventional cytotoxic agents like paclitaxel and anthracyclines, their efficacy in effecting a curative response for TNBC remains suboptimal, concurrently giving rise to pronounced adverse effects. In contrast, LAS exhibits good inhibitory activity against TNBC both in vivo and in vitro. Additionally, LAS shows no toxicity when applied in vivo. Furthermore, LAS inhibits the PI3K/Akt/mTOR and STAT3 pathways, which are two pivotal signaling pathways associated with TNBC development. Although deeper mechanisms and prognostic roles of LAS in TNBC need to be explored in the future, our findings provide a preliminary basis for evaluating it as a promising therapeutic candidate for TNBC.

## Figures and Tables

**Table 1:** IC_50_ values (μM) of LAS in human breast cancer cell lines.

Compound	Cell Line	24 h	48 h	72 h
LAS	MDA-MB-231	5.43	3.37	2.9
	MDA-MB-468	3.42	1.84	1.6
	MCF7	8.35	5.69	5.16
	MCF-10A	25.84	6.69	5.95
Oridonin	MDA-MB-231	23.38	18.96	16.79

## Data Availability

The data presented in this study are available from the corresponding author upon reasonable request.

## Supplementary Materials

The following supporting information can be downloaded at: https://www.mdpi.com/article/10.3390/molecules28237701/s1, Figure S1: cell viability of MCF-10A was measured by MTT assay after LAS treatment; Figure S2: LAS induced cell cycle arrest in MDA-MB-468 cells; Figure S3: LAS induced apoptosis and DNA damage in MDA-MB-468 cells; Figure S4: LAS inhibited the migration and invasion of MDA-MB-468 cells; Figure S5: LAS inhibited PI3K/Akt/mTOR pathway and STAT3 in MDA-MB-468 cells.
