# Supplementary material for: In Vitro and In Vivo Anti-Cancer Activity of Lasiokaurin in a Triple-Negative Breast Cancer Model
Source: Molecules. 2023 Nov 22;28(23):7701. doi: 10.3390/molecules28237701 (PMC10707582; doi:10.3390/molecules28237701)
Supplement: Supplementary file 1 [file molecules-28-07701-s001.zip › molecules-2528662-supplementary.pdf]

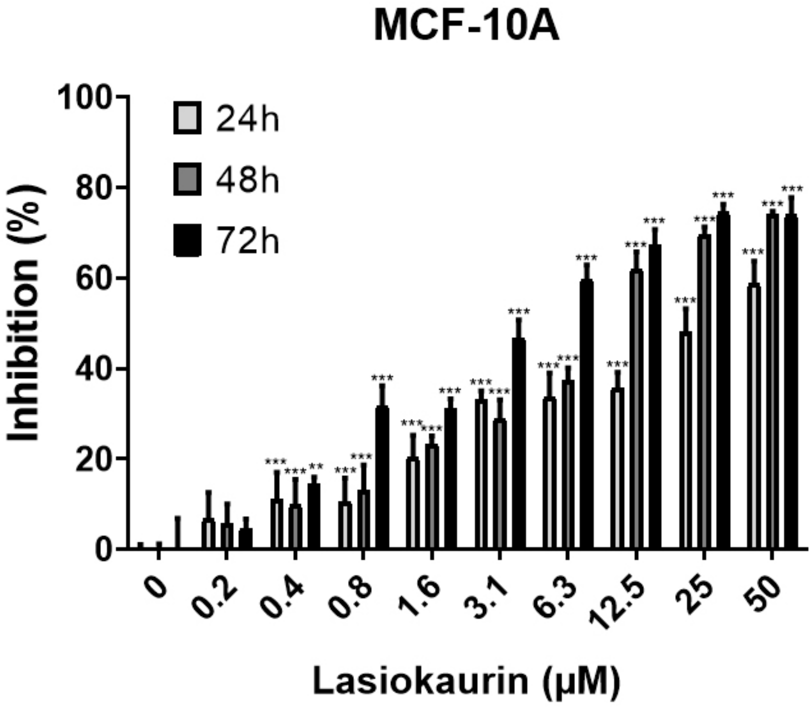

Supplementary Figure S1. Cell viability of MCF-10A was measured by MTT assay after LAS treatment. Data are presented as means ± SEM from three independent experiments. * $P < 0.05$, ** $P < 0.01$, *** $P < 0.001$, compared to control.

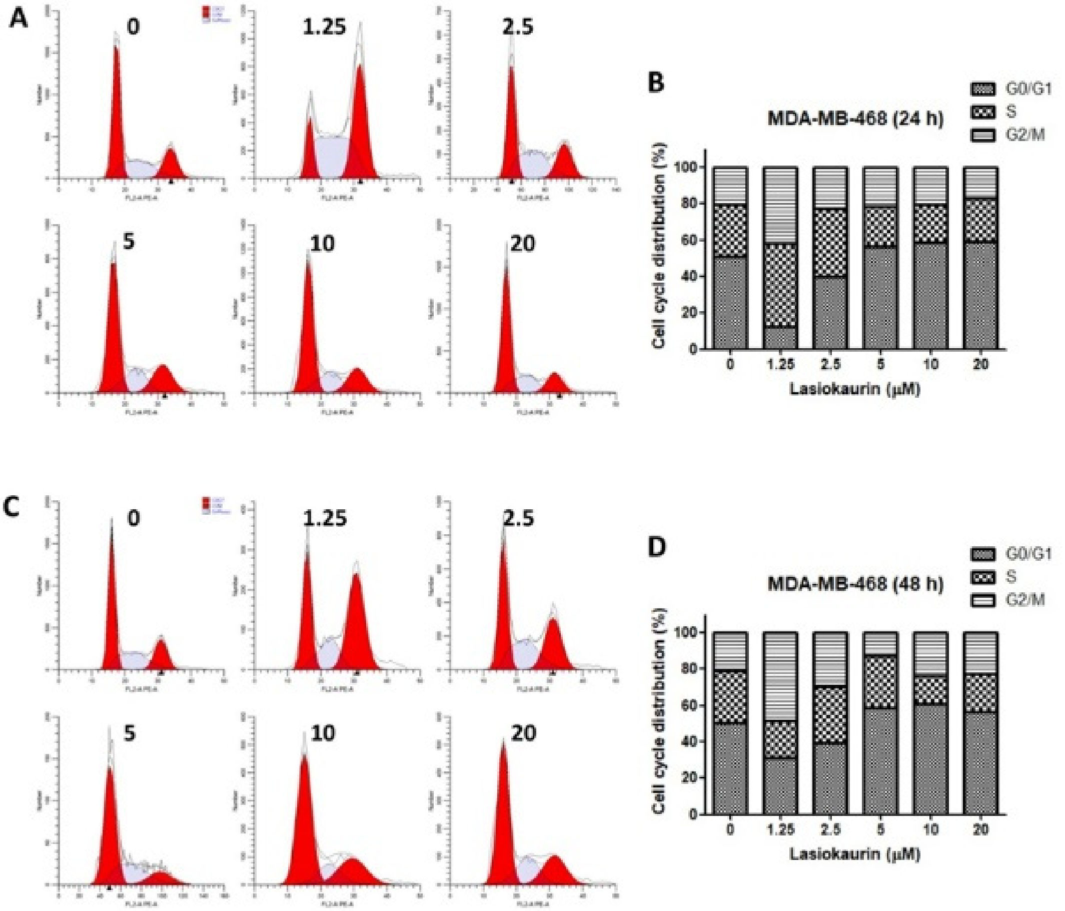

Supplementary Figure S2. Laskaurin induced cell cycle arrest in MDA-MB-468 cells. MDA-MB-468 cells were stained with PI after laskaurin treatment and the cell cycle analyzed by flow cytometry. Representative DNA fluorescence histograms of cell cycle distribution after 24 h (**A**) and 48 h (**C**) treatment were presented. Bar charts showed the percentage of different phases after 24 h (**B**) and 48 h (**D**) treatment.

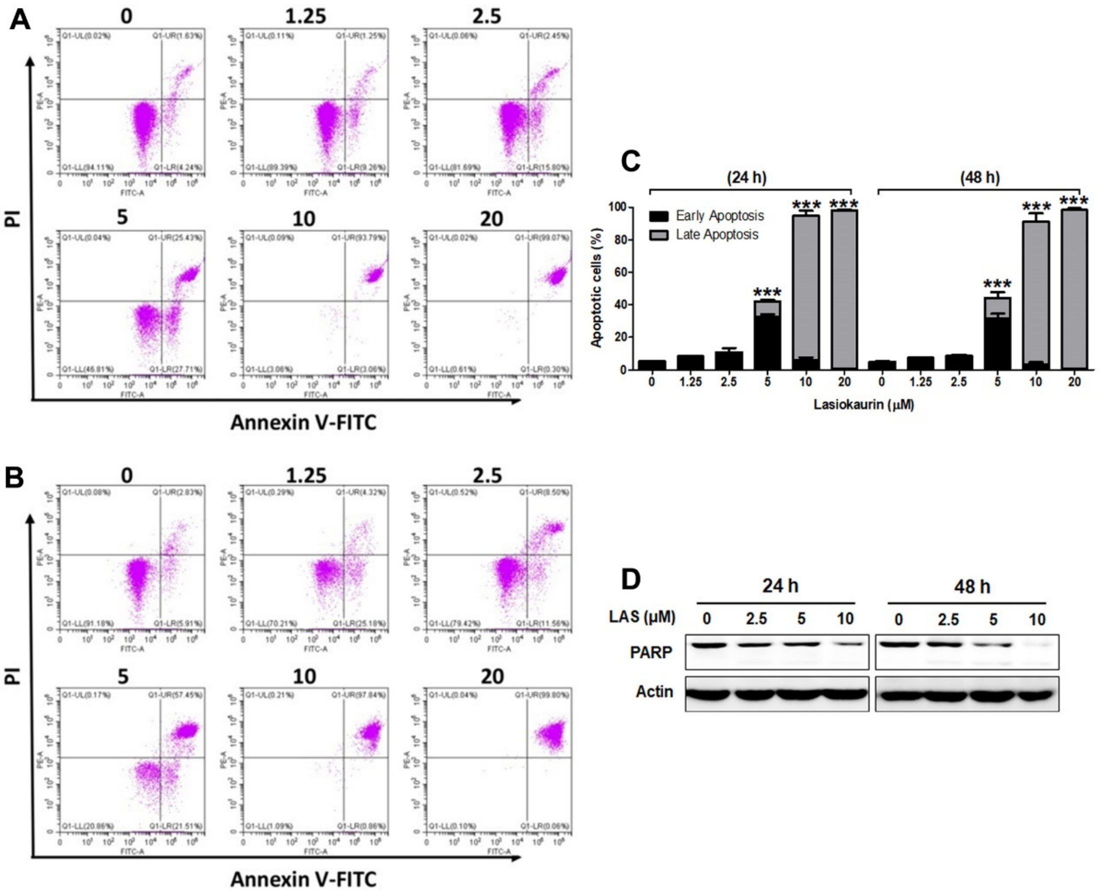

Supplementary Figure S3. Laskaurin induced apoptosis and DNA damage in MDA-MB-468 cells. MDA-MB-468 cells were treated with laskaurin for 24 h (**A**) and 48 h (**B**), stained with Annexin V-FITC/PI and cell apoptosis was analyzed by flow cytometry. (**C**) Representative flow cytometry Annexin V/PI data. * $P < 0.05$, ** $P < 0.01$, *** $P < 0.001$, compared to control. (**D**) Cell extracts were prepared from MDA-MB-468 cells and immunoblotted with the indicated antibodies. β-Actin was used as an internal control.

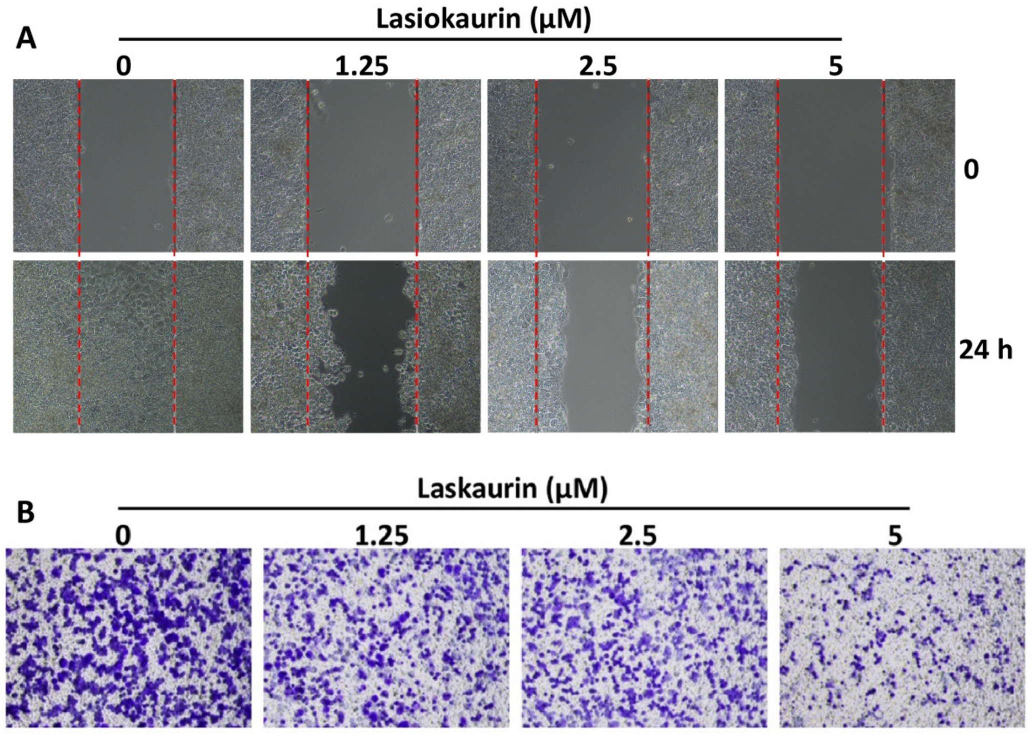

Supplementary Figure S4. Laskaurin inhibited the migration and invasion of MDA-MB-468 cells. (**A**) Cell migration was measured by wound-healing assay. (**B**) Cell invasion ability was assessed by transwell invasion assay.

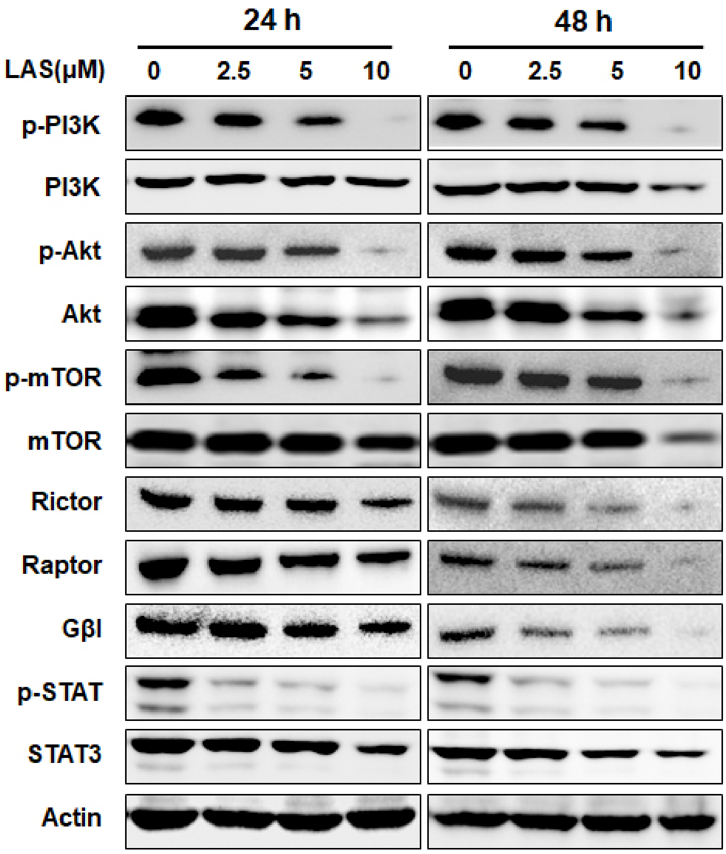

Supplementary Figure S5. Laskaurin inhibited PI3K/Akt/mTOR pathway and STAT3 in MDA-MB-468 cells. MDA-MB-468 cells were treated with laskaurin at concentrations of 1.25, 2.5, 5 μM for 24 or 48 h. Cell pellets collected and immunoblotted with the indicated antibodies. β-actin was used as an internal control.
